# Design of an on-chip wavelength conversion device assisted by an erbium-ytterbium co-doped waveguide amplifier

**DOI:** 10.1007/s12200-024-00118-2

**Published:** 2024-06-04

**Authors:** Chen Zhou, Xiwen He, Mingyue Xiao, Deyue Ma, Weibiao Chen, Zhiping Zhou

**Affiliations:** 1 School of Physical Sciences, University of Science and Technology of China, Hefei, 230026 China; 2 Aerospace Laser Technology and System Department, Shanghai Institute of Optics and Fine Mechanics, Chinese Academy of Sciences, Shanghai, 201800 China; 3 Hangzhou Aijie Optoelectronic Technology Co. Ltd., Hangzhou, 311400 China

**Keywords:** Silicon-based optoelectronics, Wavelength conversion, Waveguide amplifier, 2 μm band

## Abstract

In current documented studies, it has been observed that wavelength converters utilizing AlGaAsOI waveguides exhibit suboptimal on-chip wavelength conversion efficiency from the C-band to the 2 μm band, generally falling below −20.0 dB. To address this issue, we present a novel wavelength conversion device assisted by a waveguide amplifier, incorporating both AlGaAs wavelength converter and erbium-ytterbium co-doped waveguide amplifier, thereby achieving a notable conversion efficiency exceeding 0 dB. The noteworthy enhancement in efficiency can be attributed to the specific dispersion design of the AlGaAs wavelength converter, which enables an upsurge in conversion efficiency to −15.54 dB under 100 mW of pump power. Furthermore, the integration of an erbium-ytterbium co-doped waveguide amplifier facilitates a loss compensation of over 15 dB. Avoiding the use of external optical amplifiers, this device enables efficient and high-bandwidth wavelength conversion, showing promising applications in various fields, such as optical communication, sensing, imaging, and beyond.

**Graphical Abstract:**

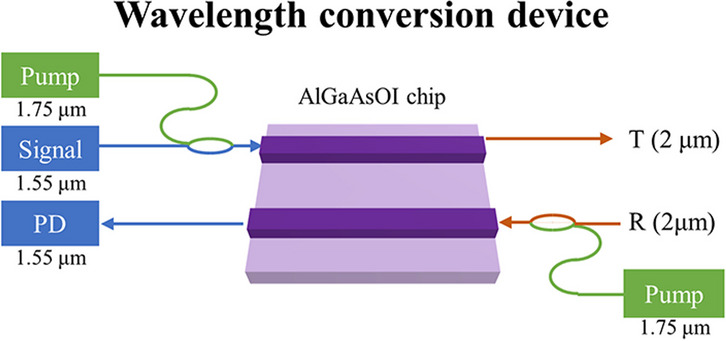

## Introduction

The 2 μm wavelength band has applications in many fields. It is paramount in gas detection due to the strong absorption spectral lines of common greenhouse gases like methane and carbon dioxide in 2 μm band, enabling sensitive gas sensing [1, 2]. Besides, in the realm of remote sensing and ranging, the 2 μm band serves as a crucial transparent window in the atmosphere with high penetrating power [3]. In addition, in optical communication, where technology is nearing the Shannon limit [4], leveraging the 2 μm band is deemed a pivotal direction for communication expansion [5]. The vast potential of the 2 μm band has garnered significant attention. Nonetheless, the absence of commercial 2 μm lasers, modulators, and detectors poses a significant challenge in constructing a comprehensive transceiver system for the 2 μm band. The development of these essential devices is often costly and time-consuming [6].

Wavelength conversion offers a viable solution to the issues [6, 7]. This process leveraging the principle of four-wave mixing (FWM) facilitates the conversion of light across various bands, which enabling 2 μm band optical transmission utilizing well-established commercial C-band transmitters and receivers [8]. High conversion efficiency in wavelength converters is paramount for the implementation of a reliable 2 μm band transceiver system [9]. Traditional methods of enhancing wavelength conversion efficiency often entail deploying external fiber amplifiers [10, 11] or increase waveguide length, consequently leading to increased device dimensions and system complexity.

In this study, we have developed an effective on-chip wavelength conversion device. The device combines a wavelength converter with AlGaAs materials, known for their high nonlinear coefficients, and a waveguide amplifier with erbium-ytterbium co-doped materials. This device facilitates the implementation of a comprehensive 2 μm band transceiver system utilizing commercial components. At the end of the transmitter, the device accepts light emissions from a C-band laser, converting them to the 2 μm band via the wavelength converter, thereby replacing the original 2 μm light source (more sophisticated technology necessitated). Similarly, at the end of the receiver, the device receives an external 2 μm optical signal, which is then reconverted to the C-band through the wavelength converter and outputted into a C-band detector for efficient detection, thereby circumventing the need for 2 μm detectors (inefficient and detector options limited). Furthermore, the integration of erbium-ytterbium co-doped waveguide amplifiers within this device serves a dual purpose—compensating for energy losses in wavelength conversion and filtering out residual idle and pump lights post wavelength conversion. Our design integrates different semiconductor materials and innovatively achieves both wavelength conversion and waveguide amplification on a single chip. By using waveguide amplifiers to compensate for power loss, without the need for external optical fiber amplifiers, this self-contained wavelength conversion device offers an integrated, compact, low consumption and efficient solution for achieving wavelength conversion. Avoid the preparation of 2 μm band devices, our design catalyzes the advancement of high-performance 2 μm band transceiver systems, and advancing the application of 2 μm band in communication, sensing, detection and other fields.

## Structure of the wavelength conversion device

**Fig. 1:**
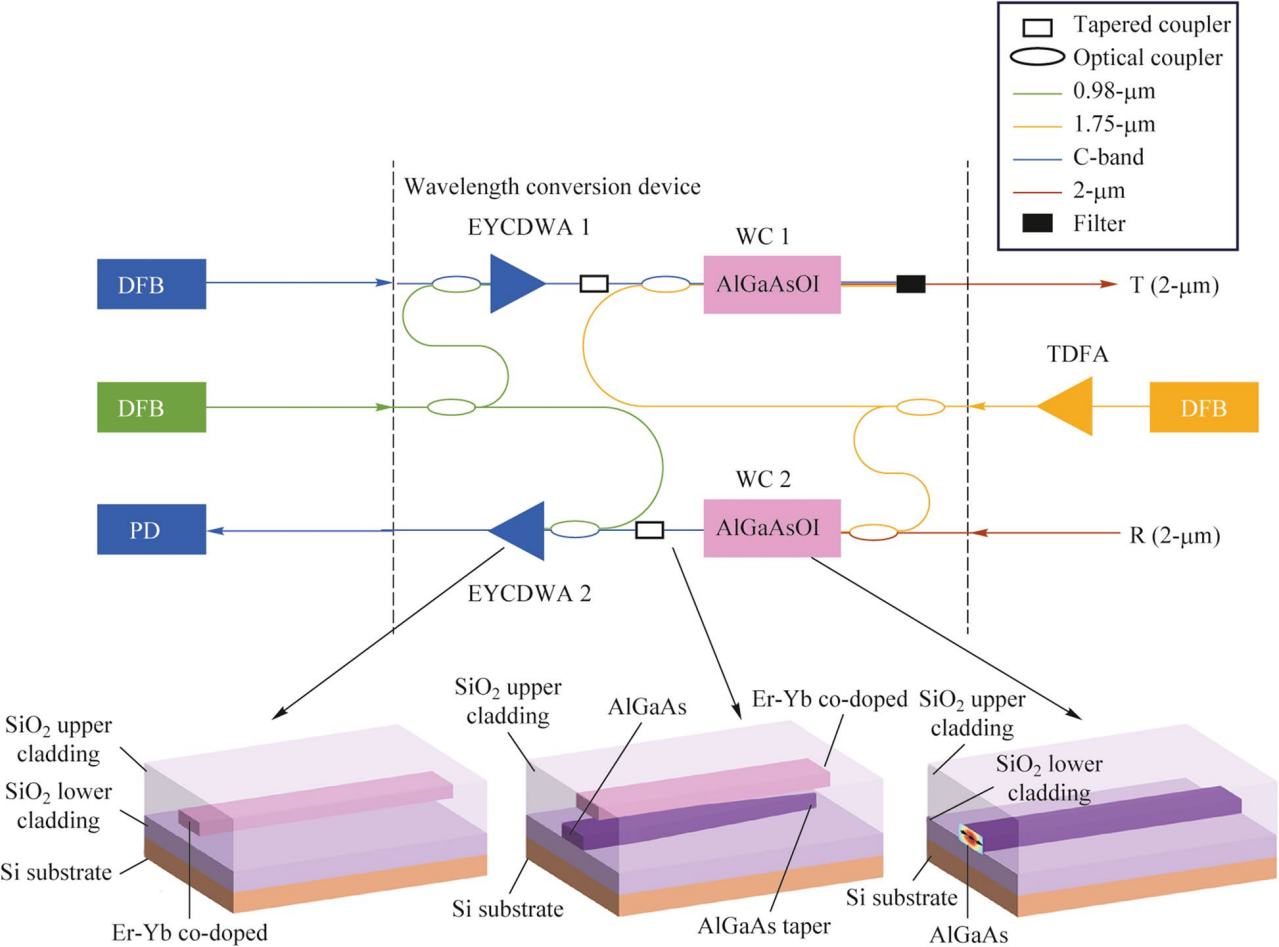
On-chip wavelength conversion device and the structure of three key component

Figure 1 depicts the device structure, comprising primarily three key components: erbium-ytterbium co-doped waveguide amplifier (EYCDWA), wavelength converter (WC), and double-layer tapered coupler. Initially, the device accepts the optical signal from the C-band laser, which undergoes amplification by EYCDWA1.

Subsequently, it is combined with pump light and fed into WC1. Here, the C-band signal is transformed into the desired 2 μm signal before being emitted. Similarly, at the receiving end, the device receives 2 μm signal light, which is reconverted to the C-band by WC2 and then amplified by EYCDWA 2. Finally, the amplified signal is outputted to a C-band detector for further processing.

### Design of the wavelength converter

Four-wave mixing is a widely used nonlinear optical technique for wavelength conversion. High efficiency in the four-wave mixing process requires the satisfaction of a phase-matching condition. In the case of degenerate FWM, the phase matching condition can be represented mathematically as [12]:

1 $$\Delta k=\Delta {k}_{nl}+\Delta {k}_{l}=0,$$

where $\Delta {k}_{l}=2{\beta }_{\text{p}}-{\beta }_{\text{s}}-{\beta }_{\text{i}}$, and ${\beta }_{\text{p}}$, ${\beta }_{\text{s}}$, ${\beta }_{\text{i}}$ are the propagation constant of the pump, signal, and idler, respectively. To convert the wavelength from near 2 μm to the C-band, a larger span of frequency conversion is needed. So, the 4th-order waveguide dispersion is retained.

Then expanding the linear phase mismatch at the pump frequency, and the linear phase mismatch can be simplified to:

2 $$\Delta {k}_{l}={\beta }_{2}\Delta {\omega }^{2}+\frac{1}{12}{\beta }_{4}\Delta {\omega }^{4},$$

where $\Delta \omega =\left|{\omega }_{\text{p}}-{\omega }_{\text{s}}\right|=\left|{\omega }_{\text{p}}-{\omega }_{\text{i}}\right|$, ${\beta }_{2}$ is the 2nd-order waveguide dispersion, and ${\beta }_{4}$ is the 4th-order waveguide dispersion. To minimize the phase mismatch, $\Delta {k}_{l}=0$, we have:

3 $$\begin{array}{c}\Delta \omega =\pm \sqrt{-12\left({\beta }_{2}/{\beta }_{4}\right)}.\end{array}$$

If the anomalous dispersion method is adopted, we will obtain the phase matching points in both the near-pumping wavelength region and the far-pumping wavelength region, and the phase matching point in the near-pumping wavelength region will produce quantum noise, which will affect the pumping power. By setting the pump wavelength to the normal dispersion region of near zero and making it have negative 4th-order dispersion, we can obtain phase matching only in the far pump wavelength region, reducing the loss caused by noise.

The dispersion properties of the waveguide are:

4 $$\begin{array}{c}{\beta }_{n}=\frac{{\text{d}}^{n}\beta }{\text{d}{\omega }^{n}} \left(n=\text{1,2},3,\dots \right),\end{array}$$

5 $$\begin{array}{c}\beta ={n}_{\text{eff}}\left(\omega \right)\frac{\omega }{c},\end{array}$$

where $\beta$ is the propagation constant of light wave with frequency *ω* in the waveguide, $n_{\text{eff}}$ is the effective refractive index of the waveguide, and ${\beta }_{n}$ is the dispersion coefficient of order *n*.

The relationship between effective refractive index and frequency of waveguide is obtained by simulation, after substituting Eq. (5), the propagation constant is fitted to a higher order polynomial, then the 2nd-order waveguide dispersion and 4th-order waveguide dispersion of waveguides can be obtained by Eq. (4).

In the degenerate four-wave mixing process, in addition to the dispersion characteristics of the waveguide, the nonlinear coefficient of the waveguide is also an important factor affecting the nonlinear action strength. The nonlinear coefficient of waveguide is [13]:

6 $$\begin{array}{c}\gamma =\frac{2\uppi {n}_{2}}{\lambda {A}_{\text{eff}}},\end{array}$$

7 $$\begin{array}{c}{A}_{\text{eff}}=\frac{{\left({\iint }_{-\infty }{\left|E\left(x,y\right)\right|}^{2}\text{d}x\text{d}y\right)}^{2}}{{\iint }_{-\infty }{\left|E\left(x,y\right)\right|}^{4}\text{d}x\text{d}y},\end{array}$$

where ${A}_{\text{eff}}$ is the effective mode area, $\lambda$ is the wavelength of light, and $E\left(x,y\right)$ is the mode field distribution function.

Without considering the energy consumption of the pump light in the four-wave mixing process, the expression of the conversion efficiency $\eta$ of the signal is as follows:

8 $$\begin{array}{c}{L}_{\text{eff}}=\frac{1-\text{exp}\left(-a\cdot L\right)}{a},\end{array}$$

9 $$\begin{array}{c}G=\sqrt{{\left(\gamma {P}_{\text{P}}\right)}^{2}-{\left(\frac{\Delta k}{2}\right)}^{2}},\end{array}$$

10 $$\begin{array}{c}\eta ={\left(\frac{\gamma {P}_{\text{P}}}{G}\right)}^{2}{\text{sinh}}^{2}\left(G\cdot {L}_{\text{eff}}\right),\end{array}$$

where $\Delta k=\Delta {k}_{nl}+\Delta {k}_{l}=2\gamma {P}_{P}+\left(2{\beta }_{\text{p}}-{\beta }_{\text{s}}-{\beta }_{\text{i}}\right)$, $P_{\text{P}}$ is the power of pump, $L_{\text{eff}}$ is the effective waveguide length, $a$ is the loss per unit length, and $L$ is the length of the waveguide.

AlGaAs is indeed a versatile material with significant potential for nonlinear processes in optoelectronics. Its large Kerr coefficient ${n}_{2}$ [14] and 2nd-order nonlinear magnetic susceptibility ${\upchi }^{\left(2\right)}$ [15] make it well-suited for applications requiring nonlinear optical effects. Additionally, the ability to adjust the refractive index by varying the proportion of aluminum allows for customization of the material’s properties to suit specific applications, enabling a broad conversion bandwidth compared to other nonlinear materials. The wide transparency window of AlGaAs in the near-infrared to mid-infrared (0.9–17 µm) range [16], further enhances its appeal for optoelectronic devices operating in these spectral regions. Its tunable bandgap energy offers the advantage of mitigating the two-photon absorption effect, ensuring that the waveguide remains unaffected when pumped at specific bands. Utilizing AlGaAs in silicon-based optoelectronics could lead to advancements in various fields, including telecommunications, sensing, and quantum optics. The material’s unique combination of properties makes it a promising candidate for enabling innovative optoelectronic devices with enhanced performance.

**Fig. 2:**
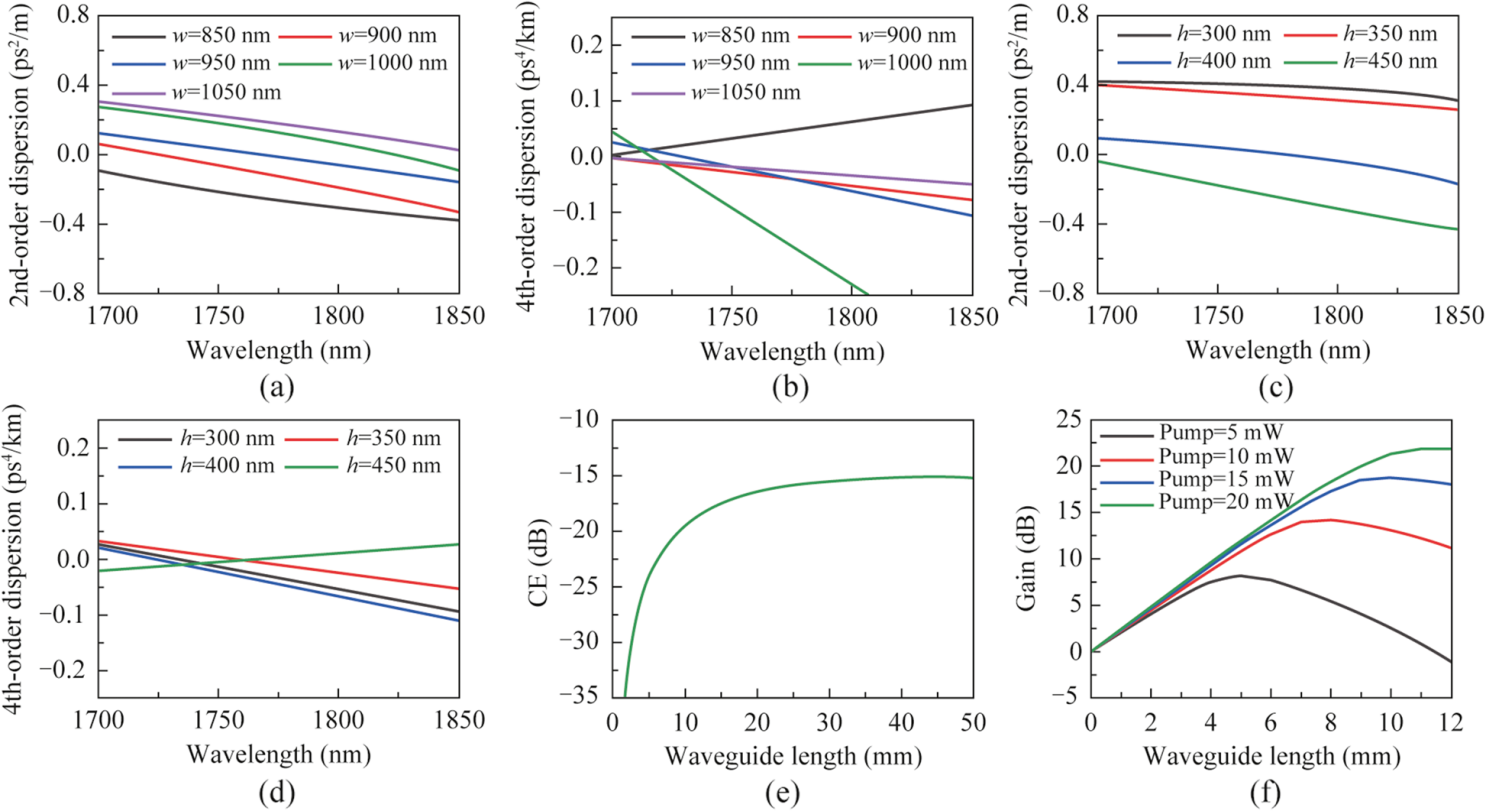
Relationship between wavelength and dispersion of **a** 2nd-order and **b** 4th-order with different waveguide width; the relationship between wavelength and dispersion of **c** 2nd-order and **d** 4th-order with different waveguide height. **e** Relationship between the waveguide length of the wavelength converter and the conversion efficiency (CE). **f** Relationship between the waveguide length of the waveguide amplifier and the gain

AlGaAsOI (AlGaAs-on-insulator) has garnered attention for its potential to maximize the benefits of AlGaAs materials as an effective nonlinear platform. In contrast to traditional AlGaAs waveguides, the AlGaAsOI platform offers a high refractive index contrast with the silica substrate. This contrast strengthens the binding of waveguides to light waves and facilitates the design and control of waveguide dispersion. Additionally, the AlGaAsOI platform boasts lower transmission losses, thereby minimizing loss during transmission [17–21]. We adjust the width and height of the waveguide. Figure 2a–d describe the relationship of the wavelength and the dispersion coefficient of the waveguide. With the increase of the waveguide width, the 2nd-order dispersion coefficient of the waveguide gradually increases. With the increase of the height of the waveguide, the 2nd-order dispersion coefficient of the waveguide gradually decreases. At the same time, in order to make the idle wavelength slightly higher than 2 μm after the four-wave mixing conversion, in the case of the input light is C-band, we need to make the pump wavelength near 1.76 μm. From this, we can conclude that when the width of the waveguide is 0.95 μm and the height of the waveguide is 0.95 μm, the pump light can achieve zero point of the 2nd-order dispersion coefficient between the above wavelength ranges.

When the height of the waveguide is 0.95 μm and the width is 0.40 μm, the waveguide structure has nearly zero normal 2nd-order dispersion coefficient and negative 4th-order dispersion coefficient in the pump wavelength near the 1.76 μm, which meets our requirements. Thus, according to Eqs. (6)–(10), we can derive the conversion efficiency of the wavelength converter. We choose a pump wavelength of 1.76 μm. Consider that the unit linear propagation loss of the waveguide is 1 dB/cm and the pump power of the wavelength converter is 100 mW, Fig. 2e shows the change curve of the conversion efficiency of the wavelength converter with the length of the waveguide. We can find that when the length of the waveguide is 3 cm, the conversion efficiency of the wavelength converter is about −15.54 dB.

### Design of the waveguide amplifier

**Fig. 3:**
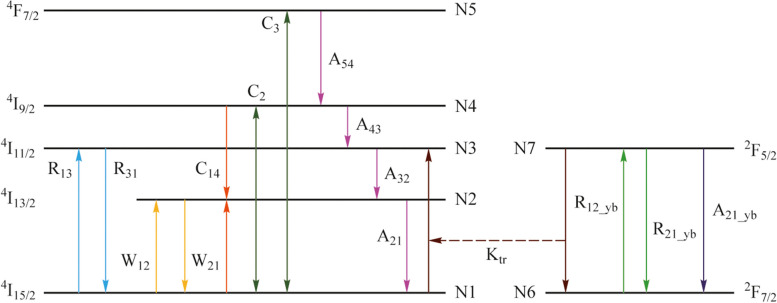
Energy level of the Er-Yb co-doped system

EYCDWA has low noise and good gain effect, and can be used to compensate for the power loss in wavelength conversion and gas detection [22–24]. The Er-Yb energy level diagram is illustrated in Fig. 3, since the absorption cross section of ${\text{Yb}}^{3+}$ is much larger than that of ${\text{Er}}^{3+}$, the ${\text{Yb}}^{3+}$ in the ground state level (N6) will transition to the first excited state level (N7) after absorbing the pump light of 980 nm, and then the ${\text{Yb}}^{3+}$ in the N7 will transfer energy to the ${\text{Er}}^{3+}$ of the N1, making it transition to the N3. Because of the short lifetime of erbium ions at N3, erbium ions at this level will rapidly decay to N2, resulting in the inversion of particle numbers between N2 and N1, thus realizing the amplification of light after wavelength conversion.

The power transmission equations in the Er-Yb co-doped system are as follows [25]:

11 $$\begin{array}{c}\frac{{\text{d}}{P}_{\text{s}}\left(Z\right)}{{\text{d}}Z}={\Gamma }_{\text{s}}\cdot {P}_{\text{s}}\left(Z\right)\cdot \left[{\sigma }_{21}\cdot {N}_{2}\left(Z\right)-{\sigma }_{12}\cdot {N}_{1}\left(Z\right)\right]\\ -{\alpha }_{\text{s}}\cdot {P}_{\text{s}}\left(Z\right),\end{array}$$

12 $$\begin{array}{c}\frac{{\text{d}}{P}_{\text{p}}\left(Z\right)}{{\text{d}}Z}={\Gamma }_{\text{p}}\cdot {P}_{\text{p}}\left(Z\right)\cdot \left[{\sigma }_{31}\cdot {N}_{2}\left(Z\right)-{\sigma }_{13}\cdot {N}_{1}\left(Z\right)\right]\\ -{\alpha }_{\text{p}}\cdot {P}_{\text{p}}\left(Z\right),\end{array}$$

13 $$\begin{array}{c}\frac{{\text{d}}{P}_{\text{ASE}}^{\pm }\left(Z\right)}{{\text{d}}Z}=\pm {\Gamma }_{\text{s}}\left({\upnu }_{j}\right)\cdot {P}_{\text{ASE}}^{\pm }\left(Z,{\upnu }_{j}\right){\cdot [\sigma }_{21}\left({\upnu }_{j}\right)\cdot {N}_{2}\left(Z\right)\\ -{\sigma }_{12}\left({\upnu }_{j}\right)\cdot {N}_{1}\left(Z\right)]\mp {\alpha }_{\text{s}}\cdot {P}_{\text{ASE}}^{\pm }\left(Z,{\upnu }_{j}\right)\\ \pm m\cdot h\cdot {\upnu }_{j}\cdot {\Gamma }_{\text{s}}\left({\upnu }_{j}\right)\cdot {\sigma }_{21}\left({\upnu }_{j}\right)\cdot {N}_{2\left(Z\right)}.\end{array}$$

The host materials of common erbium-ytterbium co-doped waveguide amplifiers include phosphate, silicate, tellurate, sulfur glass, etc. According to existing literature reports [24], we chose 5 ${\text{Er}}_{2}{{\text{O}}}_{3}$-5 ${\text{Yb}}_{2}{{\text{O}}}_{3}$-44 ${\text{La}}_{2}{{\text{O}}}_{3}$-46 ${\text{Al}}_{2}{{\text{O}}}_{3}$ as the host material of our waveguide amplifier. The material uses ${\text{Al}}_{2}{{\text{O}}}_{3}$, which can achieve high concentration of Er doping, and is modified with ${\text{La}}_{2}{{\text{O}}}_{3}$, which increase the refractive index of the material (~1.97 @1.55 μm) while reducing the formation of Er clustering. Table 1 shows the key parameters of the material.

**Table 1:** Parameters of 5 ${\text{Er}}_{2}{{\text{O}}}_{3}$-5 ${\text{Yb}}_{2}{{\text{O}}}_{3}$-44 ${\text{La}}_{2}{{\text{O}}}_{3}$-46 ${\text{Al}}_{2}{{\text{O}}}_{3}$ glass for waveguide amplifier

Parameters	Values
${\text{Er}}^{3+}$ concentration	$1.644\times {10}^{21} \text{ions/}{\text{cm}}^{3}$
${\text{Yb}}^{3+}$ concentration	$1.644\times {10}^{21} \text{ions/}{\text{cm}}^{3}$
${\text{Er}}^{3+}$ absorption cross-section at 1530 nm	$5.051\times {10}^{-21} {\text{cm}}^{2}$
${\text{Er}}^{3+}$ emission cross-section at 1530 nm	$7.303\times {10}^{-21} {\text{cm}}^{2}$
${\text{Er}}^{3+}$ absorption cross-section at 980 nm	$7.028\times {10}^{-21} {\text{cm}}^{2}$
${\text{Er}}^{3+}$ emission cross-section at 980 nm	$1.251\times {10}^{-21} {\text{cm}}^{2}$
${\text{Er}}^{3+}$ absorption cross-section at 980 nm	$1\times {10}^{-20} {\text{cm}}^{2}$
${\text{Yb}}^{3+}$ emission cross-section at 980 nm	$1\times {10}^{-20} {\text{cm}}^{2}$

According to the above parameters, Er-Yb co-doped waveguide amplifiers can be designed. Considering the actual requirements of EYCDWA and the coupling efficiency with wavelength converter, the width and height of the EYCDWA are set to 1000 and 400 nm, respectively. By solving the rate equations and power transmission equations with the 4th-order Runge-Kutta algorithm, the gain-length curve and noise-length curve of erbium-ytterbium co-doped waveguide can be obtained. With the increase of the length of the waveguide, the gain of EYCDWA gradually increases and decreases after reaching the peak, while an excessively long length may amplify the noise caused by wavelength conversion.

Figure 2f shows the relationship between the gain of the waveguide amplifier and the waveguide length with different amplifier pump power when the inanities input power is 10 μW. To offset the absence of the wavelength converter, the pump power of the waveguide amplifier was adjusted by 15 mW, the waveguide length of the amplifier was set to 10 mm. We can find that the gain of the waveguide amplifier $G_{\text{a}}$ is about 18.77 dB in this case.

### Design of the coupler

Because the waveguide layer of the wavelength converter uses AlGaAs material with refractive index *n* = 3.28, while the waveguide amplifier uses erbium-ytterbium co-doped material with refractive index *n* = 1.97. The relative refractive index difference between the two materials is so large that it is difficult for light to be coupled directly from the wavelength converter to the waveguide amplifier. Low coupling efficiency will greatly affect the overall efficiency of our chip. To solve the coupling problem of these two devices, we designed an efficient coupler for connecting the above two devices with different refractive index.

The tapered waveguide structure can change the output mode field of the waveguide and make the mode field of the two material waveguides match, thus improving the coupling efficiency.

At the same time, the double-layer structure cannot only improve the coupling efficiency, but also simplify the preparation difficulty by designing AlGaAs material and erbium-ytterbium co-doped material on different layers.

**Fig. 4:**
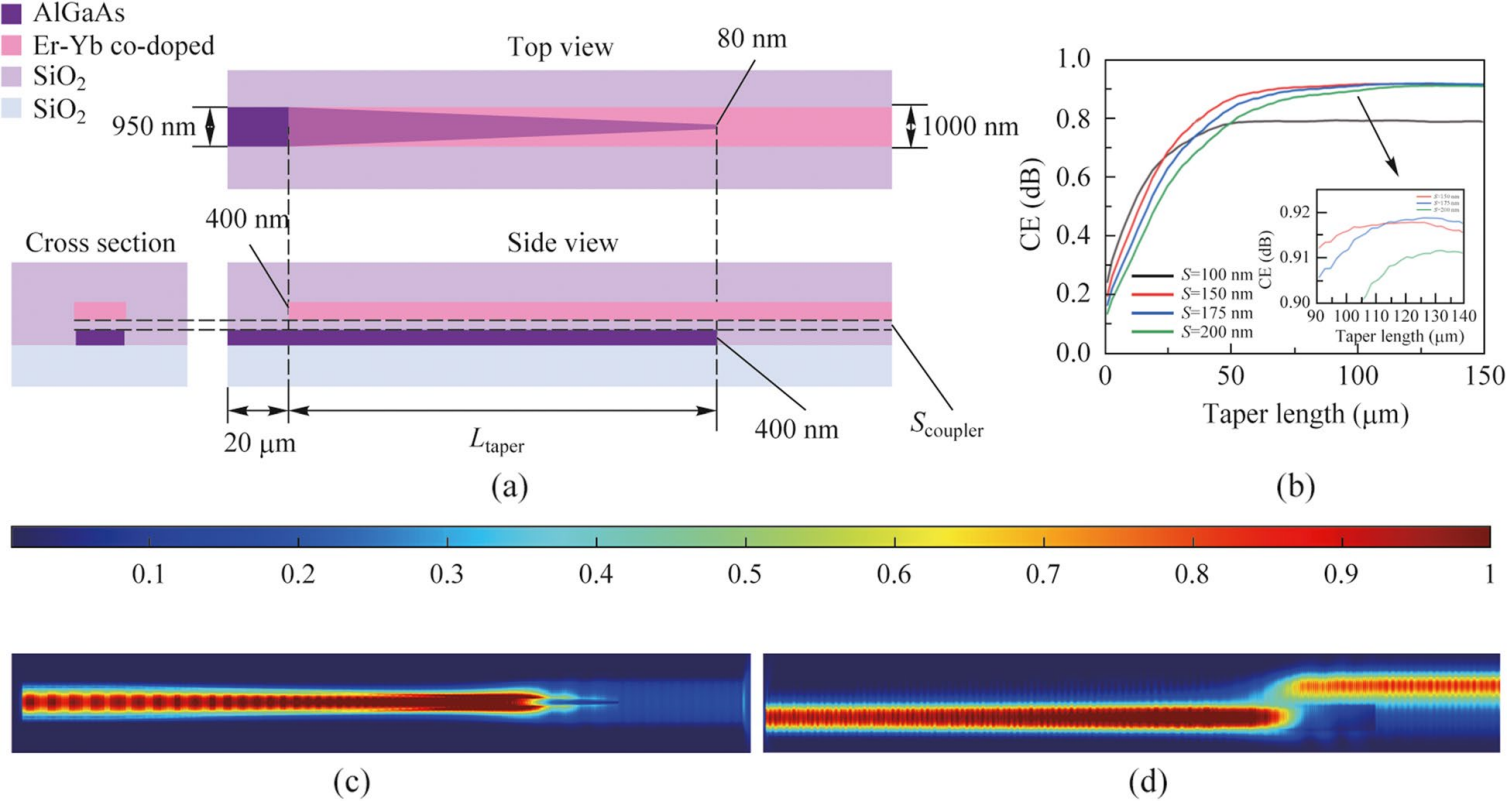
**a** Three-view drawing of the coupler. **b** Relationship between the length of inverted taper and the coupling efficiency. **c** Light field at top view. **d** Light field at side view

Figure 4a depicts three-view drawing of the tapered coupler we designed. For the rectangular waveguide, AlGaAs waveguide and Er-Yb co-doped waveguide continue the waveguide structure of wavelength converter and waveguide amplifier respectively. They parallel to each other and have a fixed height and the width of the AlGaAs waveguide gradually narrowed to the tip. The AlGaAs waveguide has a width of 950 nm and a height of 400 nm, the Er-Yb co-doped waveguide has a width of 1000 nm and a height of 400 nm. For the inverted taper AlGaAs waveguide, the length is ${L}_{\text{taper}}$, and taking into account the limitations of manufacturing technology, we set the tip width of the inverted taper structure to 80 nm. Meanwhile, there is a ${\text{SiO}}_{2}$ spacer layer with a thickness of ${S}_{\text{coupler}}$ between the AlGaAs waveguide and the Er-Yb co-doped waveguide.

Based on the above design, we simulate the inverted taper AlGaAs waveguide length ${L}_{\text{taper}}$ and the spacer layer thickness ${S}_{\text{coupler}}$. We selected the cases where the thickness of coupler spacer layer ${S}_{\text{coupler}}$ = 100, 150, 175, 200, and 250 nm, respectively. The efficiency of the coupler ${G}_{\text{cp}}$ is expressed by the quotient of the input and output power of the coupler. Figure 4b shows the relationship between the coupling efficiency of coupler and the length of inverted taper AlGaAs waveguide ${L}_{\text{taper}}$ in each case.

When the length of the inverted taper AlGaAs waveguide ${L}_{\text{taper}}$ = 114 μm and the thickness of the coupler spacer layer ${S}_{\text{coupler}}$= 175 nm, the coupling efficiency of the coupler ${G}_{\text{cp}}=91.79{\%}$. This coupling efficiency is acceptable in practical applications. Figure 4c and d show the light field at each view of the couple. As can be seen from Fig. 4c, the intensity of the light field gradually increases as the width of the waveguide decreases. The light in the tapered tip cannot be confined to the tapered waveguide, and is coupled to the rectangular waveguide. Figure 4d shows the process of coupling light from wavelength converter to waveguide amplifier.

From this, according to the above design, we can determine the final structure of the chip. As shown in the cross-section diagram of Fig. 4a, the chip is prepared in the order of Si, ${\text{SiO}}_{2}$, AlGaAs, ${\text{SiO}}_{2}$ spacer, Er-Yb co-doped material and ${\text{SiO}}_{2}$ from the bottom up. The thickness of AlGaAs, SiO_2_ spacer, and Er-Yb co-doped material is 950, 175, and 1000 nm, respectively.

## Performance results and analysis

We can calculate the efficiency of transmitting end and receiving end based on the design described earlier.

$${G}_{\text{e}}= \eta \cdot {G}_{\text{a}}\cdot {G}_{\text{cp}}.$$

**Fig. 5:**
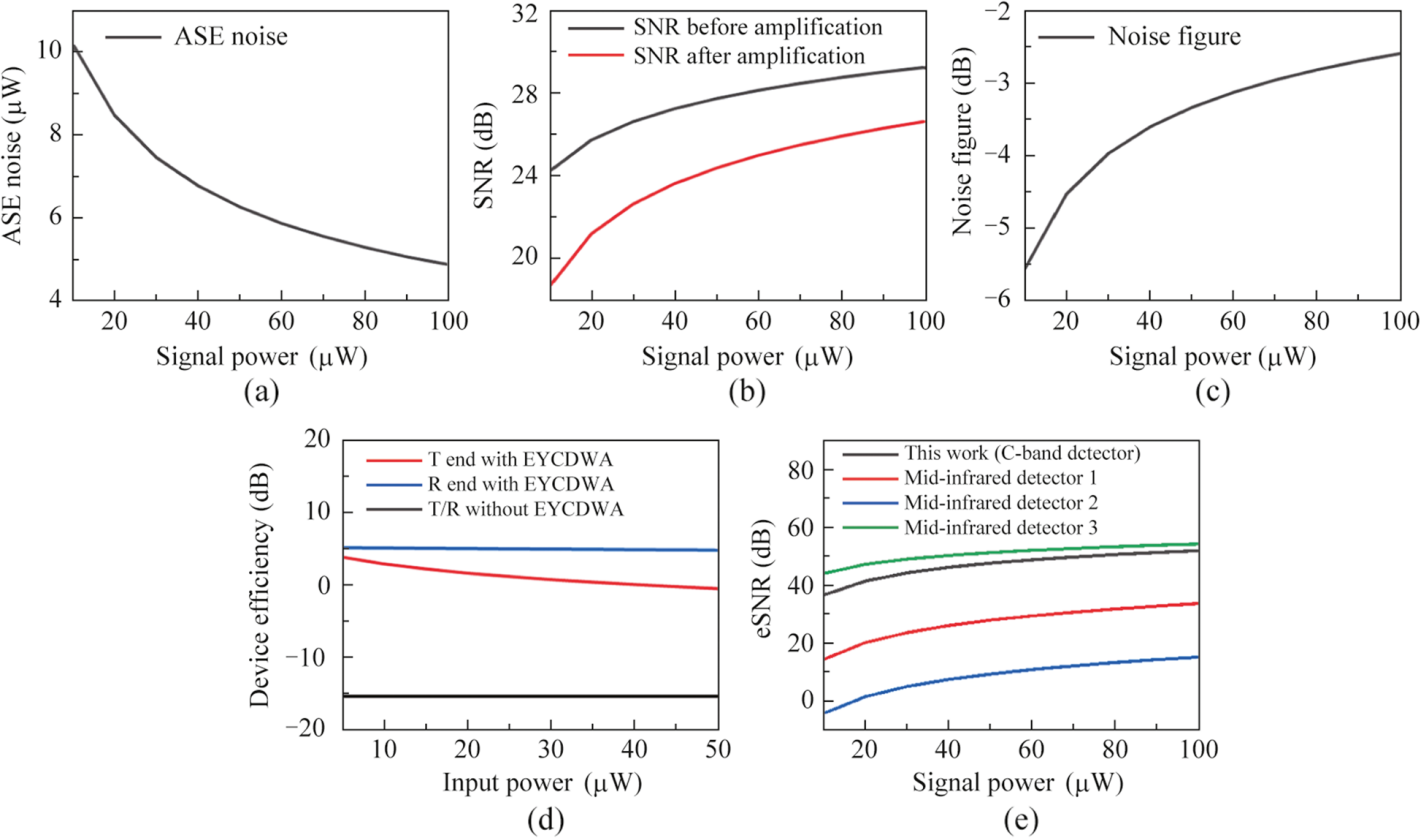
Relationship between the signal power and the **a** ASE noise, **b** SNR, **c** noise figure, **d** device efficiency, and **e** eSNR

The efficiency of each end with and without EYCDWA is depicted in Fig. 5a, revealing that our design significantly enhances efficiency. When the input power is 10 μW, the efficiency of the device is achieved 2.86 dB. The primary reason for the efficiency gap between the transmitter and receiver lies in the variance of signal power of the waveguide amplifier. In addition, the chip we designed is low-power. The total power consumption of our design comes from the pump light of the wavelength converter and waveguide amplifier. The pump power of the wavelength converter is designed to be 100 mW, and the pump power of the waveguide amplifier is 15 mW. At the same time, since there is a wavelength converter and a waveguide amplifier at both the output and input ends, the total power consumption of our chip is 230 mW. Table 2 summarizes the existing research on conversion device. As can be seen, this work achieves high conversion efficiency with low power consumption obviates the requirement for external optical fiber amplifiers.

**Table 2:** Existing research on wavelength conversion device

On-chip CE (dB)	Power consumption (dBm)	Material	Fiber amplifiers	Year	Ref.
−17.5	24.5	Si	Needed	2014	[26]
−24.5	22.96	AlGaAs	Needed	2022	[6]
−10	24	TF-PPLN	Needed	2022	[27]
−0.6	30.9	${\text{Si}}_{3}{\text{N}}_{4}$	Not needed	2023	[9]
2.86	23.62	AlGaAs	Not needed	2024	This work

The device designed in this paper achieves higher efficiency with the assist of a waveguide amplifier, enabling the convenient use of a C-band detector for indirect detecting the 2 μm signal. In comparison to direct detection using a mid-infrared detector, the noise in the indirect detection process can be attributed to the wavelength conversion, waveguide amplification, and detector detection stages.

The noise generated in the wavelength conversion process is called quantum noise, the size of the quantum noise is related to the strength of the input power, and this noise will be affected by the conversion efficiency of the wavelength converter [28, 29]. For the indirect detection chip designed in this paper, the square of the quantum noise current generated by the wavelength converter is:

14 $$\begin{array}{c}{i}_{\text{qt}}^{2}=4{R}^{2}\eta {P}_{\text{in}}\frac{hv}{2}\left(2\eta +1\right){B}_{\text{e}},\end{array}$$

where $\eta$ is the conversion efficiency of the wavelength converter, *R* is the responsivity of the detector, ${P}_{\text{in}}$ is the power of the input, ${B}_{\text{e}}$ is the bandwidth of the detector, $h$ is Planck’s constant, and $v$ is the pump light frequency.

For waveguide amplifiers, the squared of initial noise current and the input power in the amplification process are as follows:

15 $$\begin{array}{c}{i}_{\text{pa}}^{2}={i}_{\text{qt}}^{2},\end{array}$$

16 $$\begin{array}{c}{P}_{\text{ps}}={\eta \cdot P}_{\text{in}}.\end{array}$$

By bringing this initial noise and power into Eqs. (11)–(13), the total quantum noise current square ${i}_{\text{ASE}}^{2}$ and the magnification of the waveguide amplifier ${G}_{\text{a}}$ can be calculated. Then, we can calculate the signal-to-noise ratio and the noise figure of the waveguide amplifier:

$$\begin{array}{c}\text{SNR}=10{\text{log}}_{10}\left(\frac{{P}_{\text{signal}}}{{P}_{\text{noise}}}\right),\\ {N}_{\text{f}}=\Delta \text{SNR}.\end{array}$$

Figure 5b–d show the relationship between the signal power at the receiving end and the ASE noise, SNR, noise figure.

The noise generated in the detection process is mainly divided into the shot noise of the input light and the inherent noise of the detector. Among them, the shot noise will change with the process of wavelength conversion and waveguide amplification, the square of the noise current is:

17 $$\begin{array}{c}{i}_{\text{sh}-\text{in}}^{2}=\left(2qR\left( { {G}_{\text{e}}\cdot P}_{\text{in}}\right)\right)\cdot {B}_{\text{e}},\end{array}$$

18 $$\begin{array}{c}{i}_{\text{int}}^{2}={R}^{2}\cdot {\text{NEP}}^{2}\cdot {B}_{\text{e}}.\end{array}$$

From this, we can conclude that the square of the noise current of the gas detection chip designed in this paper is:

19 $$\begin{array}{c}{i}_{\text{indirect}}^{2}={i}_{\text{ASE}}^{2}+{i}_{\text{int}}^{2}+{i}_{\text{sh}-\text{in}}^{2}.\end{array}$$

Signal-to-noise ratio is the ratio of signal power and noise power, and the electrical signal-to-noise ratio of the detection chip can be summarized as:

20 $$\begin{array}{c}{\text{eSNR}}_{\text{indirect}}=10{\text{log}}_{10}\left(\frac{{R}^{2}\cdot {\left( { {G}_{\text{e}}\cdot P}_{\text{in}}\right)}^{2}}{{i}_{\text{indirect}}^{2}}\right).\end{array}$$

The eSNR calculation of direct detection is relatively simple, and its noise only includes shot noise and inherent noise:

21 $$\begin{array}{c}{i}_{\text{int}}^{2}={R}^{2}\cdot {\text{NEP}}^{2}\cdot {B}_{\text{e}},\end{array}$$

22 $$\begin{array}{c}{i}_{\text{sh}-\text{dir}}^{2}=\left(2qR{P}_{\text{in}}\right)\cdot {B}_{\text{e}},\end{array}$$

23 $$\begin{array}{c}{i}_{\text{dir}}^{2}={i}_{\text{int}}^{2}+{i}_{\text{sh}-\text{dir}}^{2},\end{array}$$

24 $$\begin{array}{c}{\text{eSNR}}_{\text{direct}}=10{\text{log}}_{10}\left(\frac{{R}^{2}{P}_{\text{in}}^{2}}{{i}_{\text{dir}}^{2}}\right).\end{array}$$

Table 3 shows the performance parameters of several common detectors. Figure 5e illustrates the correlation between the electrical signal-to-noise ratio of each detector and the power of the received signal at the receiving end, with the C-band signal being consistent at the receiver. From the data in the figure, we can see that the indirect detection method using wavelength conversion has a higher electrical signal-to-noise ratio than most direct detection methods. The indirect detection method introduces some additional noise during the conversion and amplification processes. However, the C-band detector itself has remarkable properties, including high responsiveness and low noise equivalent power. These advantageous characteristics can compensate for the loss caused by the additional noise. At the same time, although indirect detection has a slightly lower electrical signal-to-noise ratio than a few high-performance direct detections, it is possible to circumvent the demanding operating conditions associated with mid-infrared detectors, which may require cooling. Moreover, this approach offers the benefits of near-infrared detectors, such as high-speed detection and short response times. Therefore, the use of indirect detection presents a promising alternative for effective detection applications.

**Table 3:** Parameters of commercial detectors

Material	Bandwidth (Hz)	Responsivity (A/W)	NEP (W/Hz^1/2^)	Wavelength (μm)	Requirement	Name
InAsSb	1.6 × 10^6^	0.52	149 × 10^−12^	2.05	Cooled	Mid-infrared detector 1
HgCdTe	100 × 10^6^	0.05	170 × 10^−12^	2.05	Cooled	Mid-infrared detector 2
InGaAs	90 × 10^6^	1.05	0.2 × 10^−12^	2.05	Cooled	Mid-infrared detector 3
InGaAs	600 × 10^6^	1.10	4.2 × 10^−15^	1.55	Un-cooled	C-band detector

## Conclusion

In summary, we proposed an on-chip wavelength conversion device assisted by EYCDWA. The wavelength converter uses the AlGaAsOI waveguide, which has a high nonlinear coefficient. The waveguide achieves near-zero normal 2nd-order dispersion and negative 4th-order dispersion, resulting in an efficient conversion efficiency of −15.54 dB when the pump power of the wavelength converter is 100 mW. To compensate for the nonlinearity caused power loss, the incorporated waveguide amplifier achieves 18.77 dB amplification with a 15 mW pump power, 10 mm waveguide length and 10 μW initial input power. Moreover, a double-layer coupler with a coupling efficiency of 91.79% is designed to connect these components. By comparing with existing research, our design has higher wavelength conversion efficiency and lower power consumption. In addition, for investigating our device, we use the electrical signal-to-noise ratio (eSNR) as a key parameter and demonstrates that the indirect detection using our device offers convenient operating conditions and more efficient detection performance compared to direct detection. Overall, the device enables effective integrated wavelength conversion between the C-band and the 2 μm band, which is expected to be applied in various aspects of greenhouse gas detection, remote sensing and ranging, and communication expansion. Using wavelength conversion device bridges the gap between mid-infrared and C-band, this technique offers an efficient method for the development of high-performance mid-infrared transceiver systems.

## Data Availability

The data that support the findings of this study are available from the corresponding author, upon reasonable request.
